# Quartz-Enhanced Photoacoustic Spectroscopy Assisted by Partial Least-Squares Regression for Multi-Gas Measurements

**DOI:** 10.3390/s23187984

**Published:** 2023-09-20

**Authors:** Andreas N. Rasmussen, Benjamin L. Thomsen, Jesper B. Christensen, Jan C. Petersen, Mikael Lassen

**Affiliations:** Danish Fundamental Metrology, Kogle Allé 5, 2970 Hørsholm, Denmark; anr@dfm.dk (A.N.R.); blt@dfm.dk (B.L.T.); jbc@dfm.dk (J.B.C.); jcp@dfm.dk (J.C.P.)

**Keywords:** photoacoustics, gas spectroscopy, machine learning technique, partial least-squares regression, environmental sensors, methane, ammonia, humidity, optics, MIR lasers

## Abstract

We report on the use of quartz-enhanced photoacoustic spectroscopy (QEPAS) for multi-gas detection. Photoacoustic (PA) spectra of mixtures of water (H${}_{2}$O), ammonia (NH${}_{3}$), and methane (CH${}_{4}$) were measured in the mid-infrared (MIR) wavelength range using a mid-infrared (MIR) optical parametric oscillator (OPO) light source. Highly overlapping absorption spectra are a common challenge for gas spectroscopy. To mitigate this, we used a partial least-squares regression (PLS) method to estimate the mixing ratio and concentrations of the individual gasses. The concentration range explored in the analysis varies from a few parts per million (ppm) to thousands of ppm. Spectra obtained from HITRAN and experimental single-molecule reference spectra of each of the molecular species were acquired and used as training data sets. These spectra were used to generate simulated spectra of the gas mixtures (linear combinations of the reference spectra). Here, in this proof-of-concept experiment, we demonstrate that after an absolute calibration of the QEPAS cell, the PLS analyses could be used to determine concentrations of single molecular species with a relative accuracy within a few % for mixtures of H${}_{2}$O, NH${}_{3}$, and CH${}_{4}$ and with an absolute sensitivity of approximately 300 (±50) ppm/V, 50 (±5) ppm/V, and 5 (±2) ppm/V for water, ammonia, and methane, respectively. This demonstrates that QEPAS assisted by PLS is a powerful approach to estimate concentrations of individual gas components with considerable spectral overlap, which is a typical scenario for real-life adoptions and applications.

## 1. Introduction

Multi-gas detection devices that can detect a wide range of gasses and gas concentrations are highly attractive since they significantly enhance safety in various environments and can help enforce regulations with respect to allowable emission and concentrations of various gasses. There is an unmet need for accurate trustworthy gas detection devices [1,2,3]. Multi-gas sensors are valuable for real-life applications, where multiple gasses may be present or where gas concentrations can vary significantly, such as in process-control applications, airborne pollutants, medical diagnostics, and in general for monitoring industrial and urban emissions of greenhouse gasses [4,5,6,7,8,9,10,11]. Having a single device for detecting multiple gasses saves time during gas monitoring activities. Instead of having to use multiple detectors sequentially or wait for results from different devices, a multi-gas detection system can provide immediate and simultaneous measurements of several gasses [12,13]. This allows for quicker responses to potential gas leaks or unsafe conditions, enabling faster decision-making and remedial actions.

Optical spectroscopy is particularly useful for gas sensing, including gas concentration estimation [14,15,16]. Of the many optical spectroscopic methods developed during the past century, photoacoustic spectroscopy (PAS) [17,18,19,20,21] has attracted considerable interest due to its powerful, yet simple, trace gas detection method and capability to detect multiple gasses with a single device. The PAS method is different from other optical absorption-based methods, where the absorbed energy translates into kinetic energy, which forms an acoustic wave that can be detected with a simple pressure transducer [17,22,23]. A variant of PAS is quartz-enhanced photoacoustic spectroscopy (QEPAS), which was introduced in 2002 [23] and is today an established spectroscopic technique [24,25,26,27,28,29,30]. For QEPAS, the generated acoustic wave is detected using a quartz tuning fork (QTF) with a high quality factor ($Q>10^{3}$ at atmospheric pressures) and an eigenfrequency matching the laser modulation frequency [23,31]. The PAS/QEPAS technique is, however, not an absolute technique and requires calibration using certified reference gas samples. Further absolute gas concentration measurements with PAS becomes highly nontrivial in complex gas mixtures, and detailed knowledge about the chemical gas composition of the complete gas matrix is needed. This entails that absolute environmental gas concentration measurements can only be achieved upon applying a correction factor to the PAS signal, which will depend on the gas matrix. For example, the presence of water vapor in a gas sample acts as a buffer for the relaxation process and thereby enhances, or diminishes, the generated sound waves [26,32,33,34,35,36]. Furthermore, in many situations, multi-gas measurements are made difficult, if not impossible, due to strongly overlapping spectra. Therefore, most experimental PAS demonstrations have been conducted using a single gas in a simple N${}_{2}$ matrix, and only recently have applications for real-life situations been addressed [13,19,26]. However, it has been demonstrated that both identification and concentration estimation can be made possible using multivariate analysis (MVA), such as partial least-squares regression (PLS) together with gas matrix correction factors [13,37]. PLS is a machine learning technique that is widely used for regression and classification tasks. PLS combines elements of both principal component analysis (PCA) and multiple linear regression to handle situations where there are high-dimensional datasets with multicollinearity.

In this work, we demonstrate QEPAS measurements of “complex” gas mixtures with 0–12,000 ppm/V water vapor (H${}_{2}$O), 2–100 ppm/V methane (CH${}_{4}$), and 50–300 ppm/V (ammonia). The measurements were all acquired in the mid-infrared (MIR) region from 2.85 μm to 3.50 μm, enabled by a home-built wavelength-tunable optical parametric oscillator (OPO). Note that our system can scan a much broader wavelength range and is thus capable of exciting more trace gasses with the same setup. To estimate the mixing ratios and concentrations, a PLS model was trained with both HITRAN spectra and experimental PAS spectra for single components and synthetic spectra of mixtures and tested on experimental acquired PAS spectra of different mixtures of gasses. The PLS algorithm was trained to estimate the gas mixture ratio. Hereby, the concentration of all gasses in the mixture can be estimated by having just one known gas concentration. The motivation for using HITRAN spectra as training data is that instead of acquiring PAS training data, which can involve measuring many calibration data of different concentrations and be time-consuming, one simply downloads spectra from the HITRAN database and trains the PLS model. Thereby, any gas mixtures (within the wavelength range available) can, in principle, be identified, and mixing ratios can be estimated.

We are not the first group to use PLS with QEPAS. The validity of the method has been shown in the literature for QEPAS and PAS spectroscopy [13,38,39,40] and was verified to establish well-fitting spectra for both two-gas mixtures and three-gas mixtures with highly overlapping spectra. However, we claim that we, for the first time, apply PLS on PAS spectra acquired in very large spectral ranges, generated by a fast ns tunable MIR-OPO, using completely unknown gas concentrations in a realistic environment. Our work should be seen as an extension of the work presented in Refs. [13,39,40]. Additional differences between our work and those of Refs. [13,39,40] are direct amplitude measurements with the QEPAS by repetition rate synchronization and the use of both HITRAN and experimental PAS spectra for training data. In our work, the spectra are acquired in a much broader wavelength range; thus, more spectral lines are used in our PLS method, which provide more information about the complete gas matrix and makes the PLS analysis very sensitive to any potential pollutants that might influence the estimation. This adds to the complexity of the analysis compared to Refs. [13,38]. In Refs. [13,38], 2–3 PLS components are used, while we used 50 PLS components, which was found to be optimal for this wavelength range and gas composition. We believe that using PLS methods in a narrow wavelength range and with only a few PLS components as in Refs. [13,38], one needs to have prior knowledge about the gas matrix under investigation, or the PLS method may result in large uncertainties. This is not the case in our analysis since most gasses present in the atmosphere have fundamental absorption bands in the scanning range of our MIR-OPO. Our method allows us to identify other molecular components the gas matrix contains and subsequently add all observed spectra to our PLS method. We noted from our PLS analysis our samples also contained CO${}_{2}$; however, it was found that CO${}_{2}$ did not have a noticeable effect on the amount of gas estimation or on the PAS dynamics. CO${}_{2}$ was therefore removed from the PLS analysis. Finally, we applied absolute calibration of the QEPAS sensor using the PLS method, taking into account molecular relaxation effects and applying PLS to a “realistic” environment with completely unknown gas concentrations and with all natural gasses present together with enriched concentrations of methane and ammonia, with 2–3 orders of magnitude difference in concentrations.

## 2. Experimental Setup

**Figure 1:**
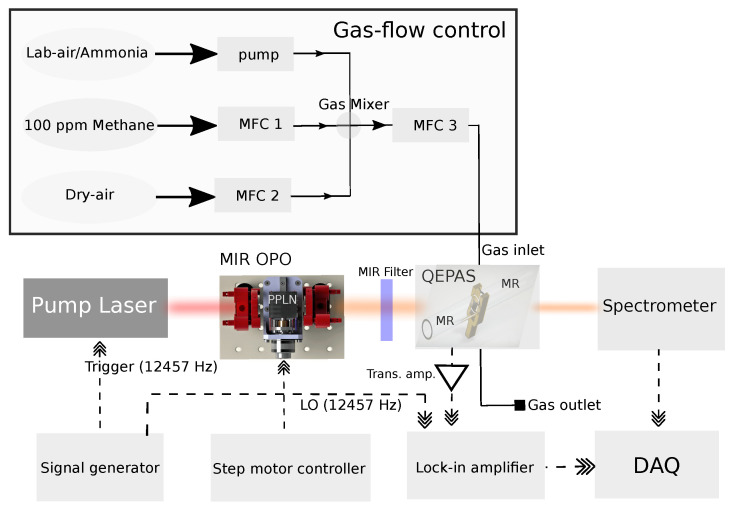
Block diagram of the main parts of the experimental setup. Actively Q-switched 1064 nm ns pump laser. QEPAS: Quartz-enhanced PAS. MIR-OPO: Mid-infrared (MIR) pulsed optical parametric oscillator. MFC: Mass-flow controller. MIR filter for removing the pump. DAQ: Signal generator for trigger signal for the 1064 nm pump laser and generating the local oscillator (both at 12,457 kHZ) for the lock-in amplifier. The downmixed signal is then acquired by an oscilloscope. The generated MIR wavelength is measured with an optical spectrometer.

The experimental setup is shown in Figure 1. The main parts in the setup include a home-built mid-infrared (MIR) nanosecond pulsed OPO, a QEPAS sensor module (ADM01, Thorlabs, Newton, NJ, USA), optical detectors for power measurement, humidity/temperature/pressure sensors, a mass-flow control system, and a lock-in amplifier and an oscilloscope for data acquisition. The MIR pump source is based on an actively Q-switched nanosecond Nd:YAG pump laser (BrightSolution, Pavia, Italy), which emits 15 ns pulses at a center wavelength of 1064 nm with a repetition rate of 12.457 kHz matching the resonant frequency of the QEPAS cell. The 1064 nm pulses are focused into a 40 mm long fan-out structured periodically poled lithium niobate crystal (PPLN) (HC Photonics) placed inside a 55 mm long linear cavity with a waist of approximately 150 μm. By translating the PPLN crystal with a step motor, MIR light from 2.85 μm to 3.55 μm with 50 mW of mean optical output power was generated. This wavelength range matches ro-vibrational lines of water (H${}_{2}$O), ammonia (NH${}_{3}$), and methane (CH${}_{4}$). More details on the MIR-OPO can be found in [26]. The QEPAS module contains a quartz tuning fork (QTF) with an eigenfrequency of $f_{0}=12,457\;\mathrm{Hz}$ and a quality factor of ~5300±50 at 1 atm [31]. The QTF is piezo-electrically active in the mechanical mode for which the two prongs oscillate 180 degrees out of phase (asymmetrical stretching mode) [23,29]. Acoustic coupling is further improved by two microresonator (MR) tubes each having a length of 12.4 mm. In- and out-coupling of the MIR light through the module happens through two BaF${}_{2}$ windows with a combined transmittance of ~0.9 [18,23,26,27,28,29].

**Figure 2:**
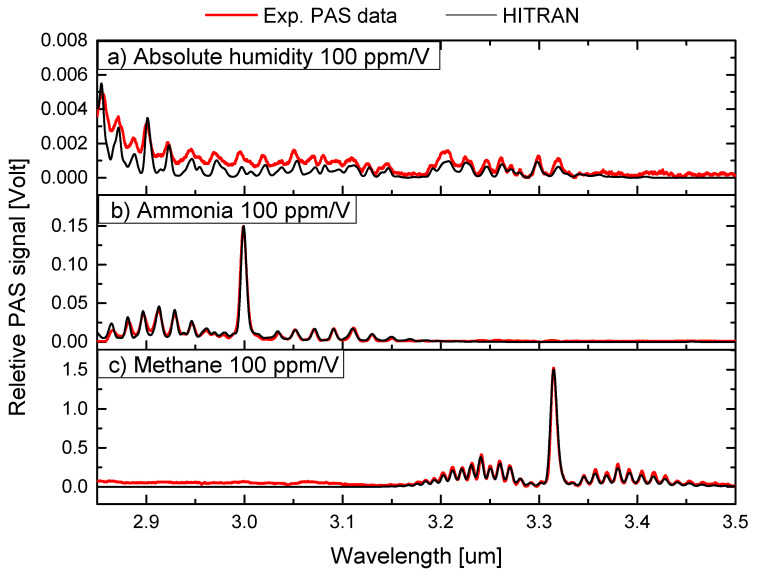
Training data for the PLS analysis for (**a**) water (H${}_{2}$O), (**b**) ammonia (NH${}_{3}$), and (**c**) methane (CH${}_{4}$). The red curves show the experimental measured PAS spectra. The black curves show the spectra from the HITRAN database convolved with a Gaussian instrument profile of 5 cm${}^{-1}$. For ammonia and methane, the R-, Q-, and P-branch are clearly observed. The y-axis is given as the measured/estimated PAS voltage and equals a concentration of 100 ppm/V for each of the three gasses. The scaling levels of the PAS signal for H${}_{2}$O and NH${}_{3}$ are estimated using 100 ppm/V of CH${}_{4}$ in N${}_{2}$.

The gas-flow control was realized using triplet mass-flow controllers (Brooks 0254, MFCs). Two MFCs were used for setting the in-flow rate of dry air/N${}_{2}$ and 100 ppmV CH${}_{4}$ in N${}_{2}$. Lab air and ammonia (NH${}_{3}$) gas flow was combined with a valve-controlled inlet that enables suction of wet laboratory air and NH${}_{3}$ into the QEPAS cell using a mini vacuum pump with variable flow rate. The humidity was measured with a commercial humidity sensor (Extech RH25, measurement accuracy 0.3%) and verified by fitting HITRAN spectra to the acquired PAS spectra, while the ammonia had a completely unknown concentration. The combined gas flow was led through a third MFC, which was used to monitor and log the total gas flow to the QEPAS module, thus estimating the concentrations of the mixed gasses with an uncertainty of ±5%. The overall gas flow was always kept at 10 mL/min through MFC3. Data processing was enabled by a lock-in amplifier receiving the electrical local oscillator signal from the active laser Q-switch with an integration time of 30 ms. The lock-in amplifier demodulates the PA output signal of the transimpedance amplifier built in the QEPAS using a 1-f configuration (i.e., amplitude modulation) [12]. The output from the lock-in amplifier was digitized using a fast 12-bit oscilloscope, and spectra in the 2.85–3.5 μm were obtained as a function of scanning time, as shown in Figure 2.

## 3. Partial Least-Squares Regression

Partial least squares (PLS) is a machine learning technique that is widely used for regression and classification tasks. PLS is a statistical method that finds a linear regression model by projecting the predicted variables and the observable variables to a new space [37]. PLS can be thought of as a hybrid between multiple linear regression and principal component analysis (PCA). In PLS, the predictor variables are first transformed into a set of latent variables using singular value decomposition. These latent variables are then used to predict the response variable in a multiple linear regression model. Thus, PLS is different from simple linear regression in that it projects the variables to a new space, where the variance is maximized along one direction in the variable hyperplane. This makes the method more powerful for fitting spectra than other fitting approaches. For example, PCA is not well suited for identifying gas mixtures in complex spectra, where one gas component completely dominates the measured spectrum, and here the PLS method performs well and can establish the actual mixture ratio with high fidelity. However, the PLS regression method is not without its limitations. It is sensitive to the scaling of the predictor variables and can be influenced by outliers in the data. It is also not suitable for cases where the predictor variables are highly correlated with the response. The general form of the PLS equation can be written as:

(1) $$\hat{Y}=XB+C,$$

where $\hat{Y}$ is the predicted response, *X* is the matrix of predictor variables, *B* is the matrix of regression coefficients, and *C* is the intercept term. The matrix *B* is obtained by performing singular value decomposition on the predictor matrix *X*. In this work, the PLS method is used to estimate the mixing ratio, thus the gas concentrations of the three gasses (H${}_{2}$O, NH${}_{3}$ and CH${}_{4}$), where they have a very high degree of spectral overlap. The PLS method used here is an implementation from the scikit-learn community [41]. A training data set was developed using either the measured single-gas PAS spectra or HITRAN spectra as reference spectra, as shown in Figure 2. The main motivation for using HITRAN spectra is that instead of acquiring PAS training data, which can be time-consuming, one simply downloads spectra from the HITRAN database to train the PLS model. This will in principle enable the estimation of any gasses (within the wavelength range available) and mixing ratios. The PLS algorithm was trained with a data set of 5000 artificial spectra composed of the three reference spectra using random ratios of the gas mixture, with a small amount of white noise added. Thus, the algorithm was trained to estimate the gas mixture ratio of the three gasses. The PAS technique is not an absolute technique, and therefore, calibration of the acquired PAS signal is required against a known gas sample. We used the obtained lock-in voltage signal for 100 ppm/V methane in N${}_{2}$ to set the expected PAS voltage level for 100 ppm/V water and ammonia. The PAS data and HITRAN data can now be used to estimate the concentration of unknown mixtures of these gasses and be used to train the PLS method. These are shown in Figure 2.

## 4. Results

The acquired PAS spectra for the training of the PLS are shown in Figure 2 together with absorption spectra from HITRAN. The HITRAN data were convolved with a Gaussian instrument profile of 5 cm${}^{-1}$. This shows that our QEPAS sensor has a spectral resolution bandwidth of 5 cm${}^{-1}$. The pressure used for these simulations was 1013 mbar and at a temperature of 25 ${}^{\circ }$C. These parameters closely resemble experimental conditions in our lab. Prior to the PLS analysis, the experimental data were post-processed to make them useful for training and validation, as well as to be compared with the PLS model trained with the corresponding HITRAN data. The raw spectral data were acquired as a function of time, and subsequently, the time x-axis was transformed into a wavelength x-axis using the Sellmeier equation for PPLN [42]. To achieve this, we merely measured the start and end wavelengths of the scan. The absolute wavelength of the probe light was measured using a calibrated optical spectrum analyzer (OSA205C, Thorlabs, Newton, NJ, USA) to establish the start and end of the wavelength x-axis. It was confirmed that the wavelength of the scans conforms very well with the Sellmeier equation. However, we found some of the acquired spectra had small deviations and nonlinear behavior of the transformation from time to wavelength, which was related to the heating effects of the PPLN crystal and acceleration–deacceleration of the stepping motor. To make the experimental data useful and give reliable predictions using both experimental PAS data and HITRAN for training, we therefore shifted the wavelength axis at the start and end to secure maximum overlap between HITRAN and experimental PAS data. Continuous measurement of the wavelength is being implemented for future work to improve this.

### 4.1. Performance and Calibration of the PLS Method

**Figure 3:**
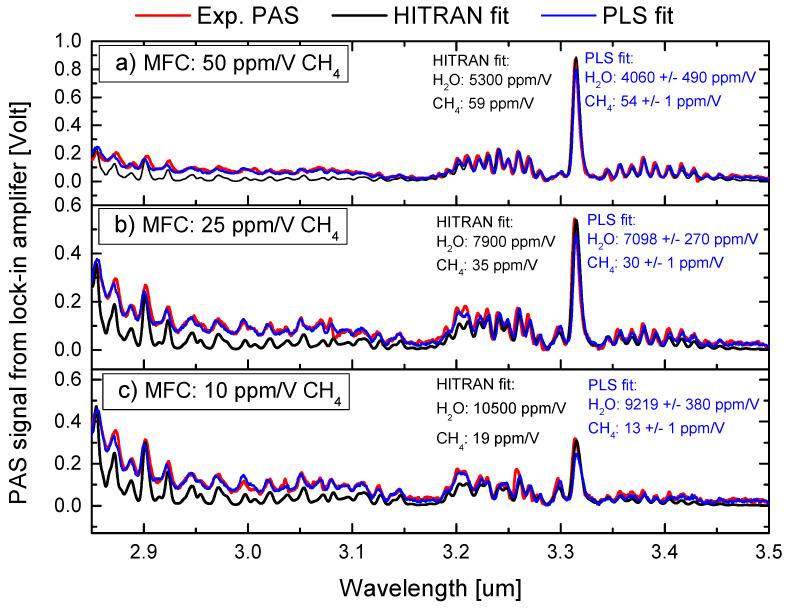
Test data containing a mixture of known water and methane concentrations. The red curves are the experimental PAS data, and the black curves are the HITRAN-fitted spectra with corresponding coefficients. The blue traces are the fitted PLS method with coefficients. The model was trained on combinations of 5000 experimental PAS spectra with superimposed Gaussian noise. The concentration of methane was controlled using the MFC and the water humidity was measured by the humidity sensor: (**a**) 50 ppm/V (±2.5 ppm) of methane and 5000 ppm/V (±250 ppm) of water humidity; (**b**) 25 ppm/V (±1.25 ppm) and 7500 ppm/V (±375 ppm) of absolute humidity; (**c**) 10 ppm/V (±0.5 ppm) of methane and 9000 ppm/V (±450 ppm) of absolute humidity.

The PAS spectra in Figure 3 show test data containing gas mixtures of water and methane with known concentrations. The ambient concentration of water in the lab air was approximately 10,400 ppm/V measured with a humidity sensor. The methane concentration with an uncertainty of ±5% was estimated using the gas flow into the QEPAS. For the data shown in Figure 3, we used flow ratios of 50/50, 25/75, and 10/90, resulting in methane concentrations of 50 (±2.5) ppm/V, 25 (±1.25) ppm/V, and 10 (±0.5) ppm/V, respectively. In Figure 3 (black traces), HITRAN data are directly fitted to the PAS data to estimate the concentrations of water and methane. The concentration of the water and methane was also estimated by the PLS algorithm trained on a linear combination of 5000 experimental training PAS spectra. The training data are shown in Figure 2a,c and superimposed with Gaussian noise to mimic experimental variations. The experimental spectra were normalized in the prediction step, which was found to significantly improve the prediction reliability of the PLS implementation. We found that both the HITRAN and PLS fitting overestimate the methane concentrations. The reason for this is that the measured PA signal is gas-matrix-dependent, meaning that PA-based trace gas sensors, while they can be extremely sensitive, can quickly become inaccurate without adequate calibration of the necessary gas matrix corrections. Most prominent, and relevant for real-life adaptation of PA sensors, is the presence of water vapor, which acts as a catalyst for the molecular relaxation process. The ro-vibrational relaxation is a result of inelastic scattering between the excited molecule and other molecules in the gas matrix. The existence of water vapor strongly mediates the relaxation process, which can result in a misleading enhancement or, in some cases, attenuation of the PA signal [26,34]. This demands that absolute environmental gas concentration measurements can only be achieved upon correction for the water content.

### 4.2. Water Correction Factors for Absolute Calibration of the PLS Method

**Figure 4:**
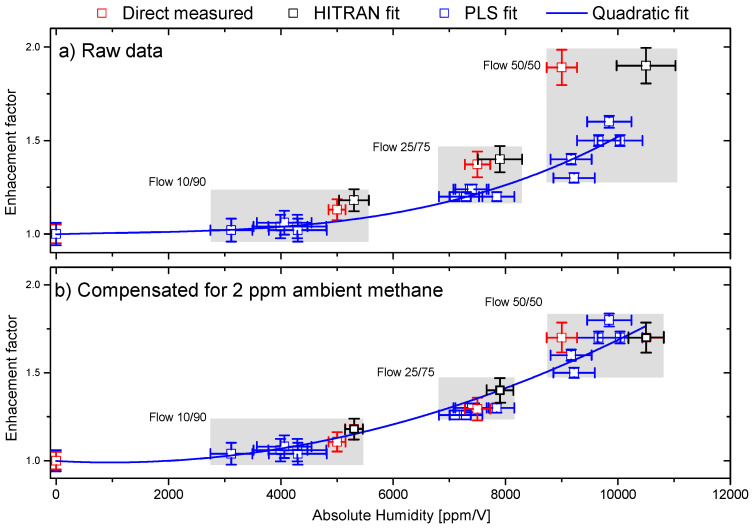
(**a**) Enhancement factor of the methane signal as a function of absolute humidity. Four flow settings (ratios between the methane and lab air) were used for the data: 100/0, 10/90, 25/75, and 50/50, respectively. Three different methods are used to estimate the enhancement factor, as shown with the color code. The shaded areas show the uncertainty area for the estimation of water and methane concentrations across the three different methods. The fitted blue curves are quadratic functions. (**b**) Same data as in (**a**) compensated for 2 ppm of ambient methane. Note that the uncertainty area is decreased by compensation for the ambient methane.

Figure 3 demonstrates how the presence of water molecules enhances the PA signal of methane. In the literature, the enhancement has previously been demonstrated to be both gas- and wavelength-dependent, and the factor does not necessarily seem to be a simple linear function of absolute humidity, as also demonstrated here [26]. The measured enhancement factor is depicted in Figure 4a as a function of absolute humidity. The humidity was measured by a relative humidity sensor (Extech RH25, measurement accuracy 3%). Using a high-end calibrated humidity sensor, we confirmed that the measurement accuracy is indeed within 3%. We found that while acquiring the experimental PAS data, the humidity fluctuates slightly in our lab between 9000 ppm/V and 12,000 ppm/V. We applied three different methods for estimating the water enhancement factor: by fitting directly with the PAS voltage signal from the lock-in amplifier followed by normalization to the voltage level for 100 ppm/V-N${}_{2}$ concentration, by fitting with HITRAN data, and by using the PLS training methods. For this, we used two models: one trained with 5000 experimental PAS spectra and a second model trained with 2500 experimental spectra + 2500 HITRAN spectra. We found that within the range of humidity used in this work, the enhancement factor shows quadratic growth, and the PA signal for methane is seen to be enhanced by more than a factor of 1.75 as a result of an absolute humidity level of approximately 10,000 ppm/V. Note that similar quadratic behavior has also been demonstrated for ammonia detection with PAS [43]. The standard deviation (STD) across all four fitting methods is for a 10/90 flow ratio: 711 ppm/V for water and 2.5 ppm/V for methane, for a 25/75 flow ratio: 290 ppm/V for water and 2.6 ppm/V for methane, and for a 50/50 flow ratio: 540 ppm/V for water and 2.9 ppm/V for methane. Figure 3 and Figure 4a strongly underline that absolute PA-based concentration measurements in humidity conditions necessarily involve a highly sensitive measurement of the absolute humidity level. Thus, a sensitive humidity measurement can either be performed directly using the PA effect or, more conveniently, by embedding humidity sensors [26,33].

Besides estimating the water enhancement factor, we also estimated the natural abundance of methane in our lab air. The lab air was used for diluting the ammonia and methane gasses and to mimic a real-life environment with varying humidity. Not shown here, but from the comparison of HITRAN spectra with the PAS spectra, we found that the lab air also contains approximately 2 ppm/V of methane. This offset needs to be considered since the PLS method will underestimate the concentration while fitting directly with HITRAN (or the PAS voltage) will overestimate the concentration of methane. In Figure 4b, we applied the compensation and found that the STD for the estimation of methane across all fitting methods is indeed improved for all three flow cases. Explicitly, we found an STD for a 10/90 flow ratio of 1 ppm/V, for a 25/75 flow ratio of 1.2 ppm/V for methane, and for a 50/50 flow ratio of 2.5 ppm/V, respectively. Taking the ambient methane into account, the estimated concentrations in Figure 3a are as follows: for HITRAN, fitting 58 (±3) ppm/V, and for PLS, fitting 53 (±3) ppm/V; according to Figure 3b, for HITRAN, fitting 34 (±2) ppm/V, and for PLS, fitting 31 (±2) ppm/V; and according to Figure 3c, for HITRAN, fitting 17 (±1) ppm/V, and for PLS, fitting 13 (±1) ppm/V. Within the measurement uncertainties, both fitting methods are found to agree well. Thus, the estimated gas mixture ratios show good correspondence with the experimentally estimated mixtures. However, the trained PLS algorithm seems in general to have a slightly lower estimate of the concentrations compared to the direct fitting with HITRAN data. This is because the HITRAN data do not consider the background signal that the training and test data contain. This can clearly be seen using 5000 combinations of HITRAN spectra for training the PLS algorithm, where we found that the estimation becomes very inaccurate and gives concentration estimates of 8457/76 ppm/V (Figure 3a), 12,560/66 ppm/V (Figure 3b), and 15,376/50 ppm/V for water/methane (Figure 3c), respectively. Thus, to enable the direct use of HITRAN data for training the PLS algorithm and use them on experimental PAS data, one needs to baseline correct the experimental data. The baseline correction involves removing the underlying baseline signal that can obscure the true spectral features. Various mathematical algorithms, such as polynomial fitting, were employed and tested for baseline correction. The goal was to improve peak detection and enable more reliable quantification of the concentrations using the HITRAN spectra. However, in practice, we found that using the experimental PAS spectra for training always gave higher accuracy.

We notice that using different trained PLS models on the same training data gives slightly different results, as seen in Figure 4. This is due to the addition of random Gaussian noise that was superimposed on the simulated spectra. We found that for the 5 PLS models trained on experimental spectra STD of 12% for water in the 1000–5000 ppm/V range, and 4% in 7000–12,000 ppm/v, respectively. For methane, the PLS concentration estimations have a relative uncertainty with an STD of 6% at the 10 ppm/V level and an STD of 2% for concentrations higher than 20 ppm/V. However, we are confident that our PLS algorithm, together with the water correction and methane compensation, gives the correct estimates of the mixing ratios between water and methane within the uncertainties of our measurements and training of the models. In the following section, we will use the calibration of the PLS algorithm to estimate the unknown mixtures of water, ammonia, and methane.

### 4.3. Performance of the PLS Method on Unknown Mixtures of Water, Ammonia, and Methane

**Figure 5:**
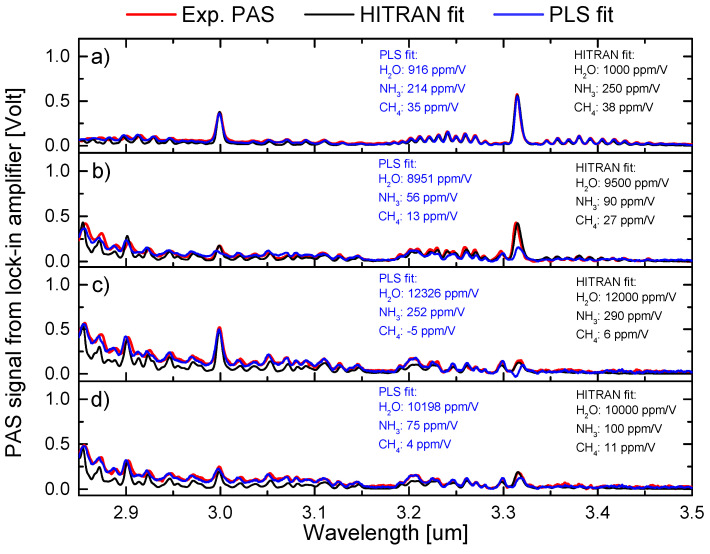
Test data for the PLS analysis of unknown concentrations of water, ammonia, and methane. The red traces are the experimental PAS spectra, and the black are the HITRAN spectra. The HITRAN spectra are fitted with the coefficients shown in black typing. The blue traces are the fitted PLS method using the coefficients shown in blue typing. The PLS model was trained on combinations of 5000 experimental PAS spectra with a superimposed Gaussian noise (0.01).

**Figure 6:**
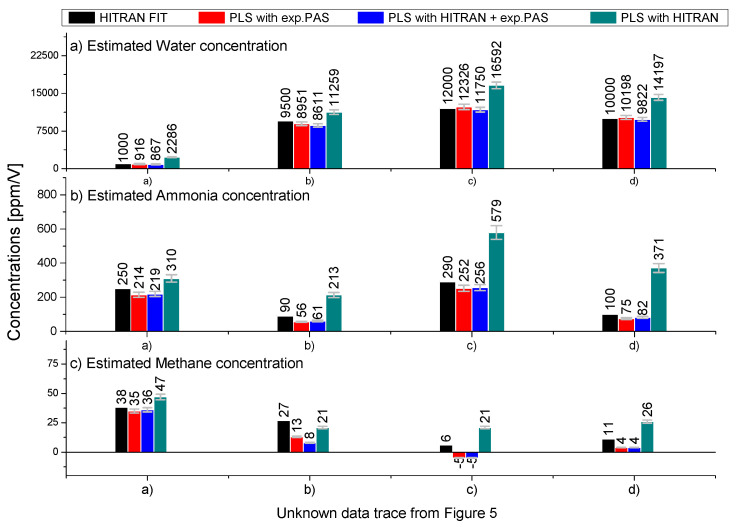
Summary of the different PLS models for estimating the unknown concentrations of (**a**) water, (**b**) ammonia, and (**c**) methane. The black columns are the fitted coefficients for the HITRAN spectra for comparison. Red columns: PLS trained on experimental PAS spectra. Blue columns: mix of HITRAN and experimental spectra. Green columns: HITRAN spectra. The error bars are given by the estimated measurement and systematic uncertainties.

The PAS spectra shown in Figure 5 depict the test data containing unknown mixtures of water, ammonia, and methane. As can be seen, the mixtures with the three gas components have high spectral overlap. In the following, we make a benchmark test of the relative accuracy of the PLS method compared with direct fitting with HITRAN spectra. The two main effects that can influence the performance and accuracy of the PLS method are high concentration of water relative to ammonia and methane concentrations and deviations in the wavelength axis compared to the training spectra. The four gas mixtures displayed in Figure 5 were chosen as extreme cases to evaluate the performance of the PLS algorithm. The PLS method was again implemented using a standard training–test approach. The training data set was built starting from the three reference spectra for water, ammonia, and methane, as shown in Figure 2. The data set was then expanded by means of calculating a linear combination of 5000 reference spectra by superimposing Gaussian noise distributions. Based on the above calibration of the PLS method for the known concentrations we can estimate the concentrations of the unknown mixtures. We assume that the water enhancement factor of ammonia has a similar behavior as methane. Figure 5a shows data for a low concentration of water and relatively similar PAS signal strength for ammonia and methane with minimal deviation in the wavelength (less than 0.2 nm) axis compared to the training/HITRAN data. Keep in mind that the Q-branch peak of methane is 10.4 times higher than the Q-branch of ammonia (as shown in Figure 2). We found that the PLS method and HITRAN fitting agree very well within the measurement uncertainties, as shown in Figure 6. The overall estimation of the water, ammonia, and methane concentrations with the PLS method has an accuracy of 92%, 86%, and 92%, respectively, relative to the HITRAN fitting. In Figure 5b, the water content is increased to 9000 ppm/V, thereby diluting the concentration of ammonia and methane. In this case, we found that the relative accuracy of the estimation of water concentration increased to 94% compared to the HITRAN fitting. Meanwhile, the accuracy of the ammonia and methane concentrations decreased to 62% and 48%, respectively. This decrease in accuracy is due to the relatively low concentration of ammonia and methane. For methane, it is mainly because of the mismatch between the wavelength axis of the test spectra and the training spectra. Comparing Figure 5b with the training data in Figure 2c, we find that the experimental PAS spectra are shifted approximately 2 nm to the left in the 3.2 to 3.35 μm range. This can be seen from Figure 5b and is the reason that the PLS fitting for methane becomes very inaccurate and underestimates the presence of methane. Note that the experimental conditions are very similar for the experimental PAS spectra shown in Figure 3b, where the wavelength deviation is less than 0.2 nm. We apply the same three-gas-trained PLS methods to the data in Figure 3b and find concentrations of 7196 ppm/V, −4 ppm/V, and 30 ppm/V for water, ammonia, and water, respectively. Thus, we conclude that the main reason for the inaccuracies shown in Figure 5b is the deviation of the wavelength axis compared to the training/HITRAN spectra, and that in order for the PLS to estimate with high accuracy, the deviation in wavelength should be less than 1 nm. In Figure 5c,d, the deviation is less than 0.5 nm, and we continue with the investigation of how a very asymmetric mixing ratio affects the accuracy of the PLS method. In this case, the concentrations of ammonia and methane become relatively low compared to the water content. The water spectra will therefore dominate the trained PLS model due to the high level of spectral overlap with ammonia and methane. To test this behavior and make an estimation of the PLS method’s lower accuracy (sensitivity) limit, we increased the amount of water and ammonia, thus diluting the content of methane at the same time. This is depicted in Figure 5c, where we find that the PLS method is making a completely wrong estimation of the concentration by estimating it to be negative (−5 ppm/V). Due to this apparent wrong estimation of the methane concentration, the PLS method predicts a 3% higher concentration of water relative to the HITRAN fitting. This fitting behavior is in general very different from the fitting behavior that we have seen in the previous cases in Figure 3 and Figure 4, where the HITRAN fitting always estimated higher concentrations. This suggests that for high concentrations of water and for relatively low concentrations of ammonia and methane, the PLS algorithm uses the ammonia and methane coefficients as a parameter to make a better fit of the water spectra. This is clearly seen from the fact that the relative precision between the PLS method and HITRAN fitting becomes higher in this case. Therefore, to estimate the accuracy and sensitivity, we diluted the concentration of water and ammonia by purging with methane, as shown in Figure 5d. It can be seen that the PLS method now gives only positive coefficients for the estimation of the concentrations. However, the estimations of ammonia and methane are still underestimated relative to the HITRAN fitting, while the water is overestimated at 2%. If we apply the enhancement and correction factors found in Figure 4 for the calibration of absolute concentrations of methane, we find a methane concentration of 3 (±2) ppm/V and 5 (±2) ppm/V for the PLS method and HITRAN fitting, respectively. Thus, the estimations of the contractions with the two methods are in good agreement with the measurement uncertainties. By applying the same water correction factor from Figure 4b to the estimated ammonia concentration, we find that the absolute concentration should be approximately 50 (±10) ppm/V and 67 (±10) ppm/V for the PLS method and HITRAN fitting, respectively. We conclude from these measurements and tests that the absolute sensitivity of the three-gas training PLS is approximately 300 (±50) ppm/V, 50 (±5) ppm/V, and 5 (±2) ppm/V for water, ammonia, and methane, respectively.

## 5. Discussion

QEPAS assisted by PLS has been demonstrated previously and reported in Refs. [13,38,40]. Compared to this work, we see our work as a natural extension, and we believe that our work is important and novel. With our analysis, we address and ask the right questions for QEPAS assisted by PLS to become a practical tool for real-life adaptations. For example, we are investigating a much wider scanning range with gas concentrations varying by two orders of magnitudes in concentration. Using the wider scanning range makes the PLS analysis become very sensitive to any potential pollutants that might influence the estimation. This results in more than 50 PLS components being needed compared to the 2–3 PLS components in Refs. [13,38,40]. However, this also adds to the complexity of the analysis, and we show how different parameters affect the analysis, such as small jitters in the wavelength x-axis, and demonstrate how it is divesting for the concentration estimation. Compared to previous work, we are testing a novel method for training and calibration of the PLS analysis and estimating the performance of the PLS method using both pure HITRAN data, experimental QEPAS data, and a mixture for training. The PLS algorithm was trained to estimate the gas mixture ratio. Thus, by proper normalization of the data, the concentration of all gasses in the mixture can then be estimated by having just one known gas concentration, in our case, methane reference gas with a concentration of 100 ppm/V. The performance of the different trained PLS models is summarized in Figure 6. The error bars are a sum of the uncertainties of the gas preparation, PAS measurement, and the PLS analysis. Three PLS models were trained, as shown by the color codes (red columns: trained with 5000 experimental PAS spectra, blue columns: with 2500 experimental PAS spectra + 2500, and green columns: 5000 HITRAN spectra). Ideally, we aimed in this pilot study to apply HITRAN spectra for the training of the PLS model. However, we found that the direct use of HITRAN becomes highly inaccurate and overestimates the concentrations, as shown in Figure 6. This is because the HITRAN spectra do not consider the relaxation mechanisms, which are both gas- and wavelength-dependent, and the potential error sources such as electrical noise and background signals that arise from stray light. The relaxation mechanisms can by proper calibration, as shown in Figure 4, indeed be considered, as also demonstrated here. The noise sources and background signals are more tedious to compensate for and are the main reason for the failure to train the PLS algorithm with only HITRAN spectra. The background signals of the experimental PAS spectra change slightly over time. However, we tried to compensate for this by realigning the MIR laser light through the QEPAS cell during a measurement series. As an alternative method, we also investigated baseline subtraction using higher-order polynomial fitting. This resulted in a slightly higher overall uncertainty in the estimation of the concentrations, and in general, we found that the direct use of the HITRAN spectra overestimated the concentration by 10–15%.

## 6. Conclusions

In conclusion, the combination of QEPAS with PLS was proven to be a strong method for the estimation of gas concentrations in complex mixtures. The mixing ratios and concentrations were estimated using PLS models trained with both HITRAN and experimental PAS spectra for single components and synthetic spectra of mixtures. The training data was built starting from the single reference spectra and was enlarged by means of simulated spectra, calculated as linear combinations of reference ones. Gaussian noise was superimposed on the simulated spectra to consider the experimental noise involved in the measurements. An absolute sensitivity, after correction for water enhancement factors and ambient methane, of approximately 500 (±50) ppm/V, 50 (±5) ppm/V, and 5 (±2) ppm/V for water, ammonia, and methane, respectively, was demonstrated.

By employing PLS to PAS sensors, concentration levels of many different target gasses can be estimated simultaneously, based on their unique spectral fingerprints. We foresee that this approach holds great promise for many different applications, such as environmental monitoring and industrial safety, providing a non-invasive and efficient solution for detecting and quantifying gasses in real-time and therefore might become a valuable tool in advancing gas detection technologies and addressing critical challenges in various fields.

## Data Availability

The data leading to the results of this work are available from the authors upon reasonable requests.

## Abbreviations

The following abbreviations are used in this manuscript:

PPLN	periodically poled lithium niobate crystal
PLS	partial least-squares regression
MFC	mass-flow controller
MIR	mid-infrared
OPO	optical parametric oscillator
PA	photoacoustic
PAS	photoacoustic spectroscopy
QEPAS	quartz-enhanced photoacoustic spectroscopy
QTF	quartz tuning fork

## References

[1] Refaat T.F., Ismail S., Koch G.J., Rubio M., Mack T.L., Notari A., Collins J.E., Lewis J., De Young R., Choi Y. (2011). Backscatter 2-μm Lidar Validation for Atomospheric CO_2_ Differential Absorption Lidar Applications. IEEE Trans. Geosci. Remote Sens..

[2] Feng S., Farha F., Li Q., Wan Y., Xu Y., Zhang T., Ning H. (2019). Review on Smart Gas Sensing Technology. Sensors.

[3] Nazemi H., Joseph A., Park J., Emadi A. (2019). Advanced Micro- and Nano-Gas Sensor Technology: A Review. Sensors.

[4] Amann A., Poupart G., Telser S., Ledochowski M., Schmid A., Mechtcheriakov S. (2004). Applications of breath gas analysis in medicine. Int. J. Mass Spectrom..

[5] Lassen M., Baslev-Harder D., Brusch A., Nielsen O.S., Heikens D., Persijn S., Petersen J.C. (2017). Photo-acoustic sensor for detection of oil contamination in compressed air systems. Opt. Express.

[6] Jongma R.T., Boogaarts M.G., Holleman I., Meijer G. (1995). Trace gas detection with cavity ring down spectroscopy. Rev. Sci. Instrum..

[7] Wilson A. (2018). Application of electronic-nose technologies and VOC-biomarkers for the noninvasive early diagnosis of gastrointestinal diseases. Sensors.

[8] Yuan Z., Bariya M., Fahad H.M., Wu J., Han R., Gupta N., Javey A. (2020). Trace-level, multi-gas detection for food quality assessment based on decorated silicon transistor arrays. Adv. Mater..

[9] Lewen Z., Zhirong Z., Qianjin W., Pengshuai S., Bian W., Tao P., Sigrist M.W. (2022). A sensitive carbon monoxide sensor for industrial process control based on laser absorption spectroscopy with a 2.3 μm distributed feedback laser. Opt. Lasers Eng..

[10] Strahl T., Herbst J., Lambrecht A., Maier E., Steinebrunner J., Wöllenstein J. (2021). Methane leak detection by tunable laser spectroscopy and mid-infrared imaging. Appl. Opt..

[11] Lang Z., Qiao S., Ma Y. (2022). Acoustic microresonator based in-plane quartz-enhanced photoacoustic spectroscopy sensor with a line interaction mode. Opt. Lett..

[12] Lamard L., Balslev-Harder D., Peremans A., Petersen J.C., Lassen M. (2019). Versatile photoacoustic spectrometer based on a mid-infrared pulsed optical parametric oscillator. Appl. Opt..

[13] Zifarelli A., Giglio M., Menduni G., Sampaolo A., Patimisco P., Passaro V.M.N., Wu H., Dong L., Spagnolo V. (2020). Partial Least-Squares Regression as a Tool to Retrieve Gas Concentrations in Mixtures Detected Using Quartz-Enhanced Photoacoustic Spectroscopy. Anal. Chem..

[14] Werle P., Slemr F., Maurer K., Kormann R., Mücke R., Jänker B. (2002). Near- and mid-infrared laser-optical sensors for gas analysis. Opt. Lasers Eng..

[15] Bogue R. (2015). Detecting gases with light: A review of optical gas sensor technologies. Sens. Rev..

[16] Hodgkinson J., Tatam R.P. (2013). Optical gas sensing: A review. Meas. Sci. Technol..

[17] Manohar S., Razansky D. (2016). Photoacoustics: A historical review. Adv. Opt. Photon..

[18] Spagnolo V., Patimisco P., Borri S., Scamarcio G., Bernacki B.E., Kriesel J. (2012). Part-per-trillion level SF_6_ detection using a quartz enhanced photoacoustic spectroscopy-based sensor with single-mode fiber-coupled quantum cascade laser excitation. Opt. Lett..

[19] Palzer S. (2020). Photoacoustic-Based Gas Sensing: A Review. Sensors.

[20] Popa C. (2019). Ethylene Measurements from Sweet Fruits Flowers Using Photoacoustic Spectroscopy. Molecules.

[21] Mikkonen T., Luoma D., Hakulinen H., Genty G., Vanninen P., Toivonen J. (2022). Detection of gaseous nerve agent simulants with broadband photoacoustic spectroscopy. J. Hazard. Mater..

[22] Westergaard P.G., Lassen M. (2016). All-optical detection of acoustic pressure waves with applications in photoacoustic spectroscopy. Appl. Opt..

[23] Kosterev A.A., Bakhirkin Y.A., Curl R.F., Tittel F.K. (2002). Quartz-enhanced photoacoustic spectroscopy. Opt. Lett..

[24] Sampaolo A., Menduni G., Patimisco P., Giglio M., Passaro V.M., Dong L., Wu H., Tittel F.K., Spagnolo V. (2020). Quartz-enhanced photoacoustic spectroscopy for hydrocarbon trace gas detection and petroleum exploration. Fuel.

[25] Tomberg T., Vainio M., Hieta T., Halonen L. (2018). Sub-parts-per-trillion level sensitivity in trace gas detection by cantilever-enhanced photo-acoustic spectroscopy. Sci. Rep..

[26] Christensen J.B., Høgstedt L., Friis S.M.M., Lai J.-Y., Chou M.-H., Balslev-Harder D., Petersen J.C., Lassen M. (2020). Intrinsic spectral resolution limitations of QEPAS sensors for fast and broad wavelength tuning. Sensors.

[27] Lassen M., Lamard L., Feng Y., Peremans A., Petersen J.C. (2016). Off-axis quartz-enhanced photoacoustic spectroscopy using a pulsed nanosecond mid-infrared optical parametric oscillator. Opt. Lett..

[28] Hayden J., Baumgartner B., Waclawek J.P., Lendl B. (2019). Mid-infrared sensing of CO at saturated absorption conditions using intracavity quartz-enhanced photoacoustic spectroscopy. Appl. Phys. B.

[29] Patimisco P., Sampaolo A., Dong L., Tittel F.K., Spagnolo V. (2018). Recent advances in quartz enhanced photoacoustic sensing. Appl. Phys. Rev..

[30] Li B., Menduni G., Giglio M., Patimisco P., Sampaolo A., Zifarelli A., Dong L. (2023). Quartz-enhanced photoacoustic spectroscopy (QEPAS) and Beat Frequency-QEPAS techniques for air pollutants detection: A comparison in terms of sensitivity and acquisition time. Photoacoustics.

[31] Friedt J.M., Carry E. (2007). Introduction to the quartz tuning fork. Am. J. Phys..

[32] Lang B., Breitegger P., Brunnhofer G., Valero J.P., Schweighart S., Klug A., Hassler W., Bergmann A. (2020). Molecular relaxation effects on vibrational water vapor photoacoustic spectroscopy in air. Appl. Phys. B.

[33] Elefante A., Menduni G., Rossmadl H., Mackowiak V., Giglio M., Sampaolo A., Patimisco P., Passaro V., Spagnolo V. (2020). Environmental Monitoring of Methane with Quartz-Enhanced Photoacoustic Spectroscopy Exploiting an Electronic Hygrometer to Compensate the H_2_O Influence on the Sensor Signal. Sensors.

[34] Müller M., Rück T., Jobst S., Pangerl J., Weigl S., Bierl R., Matysik F.M. (2022). An algorithmic approach to compute the effect of non-radiative relaxation processes in photoacoustic spectroscopy. Photoacoustics.

[35] Wysocki G., Kosterev A.A., Tittel F.K. (2006). Influence of molecular relaxation dynamics on quartz-enhanced photoacoustic detection of CO_2_ at *λ*= 2 μm. Appl. Phys. B.

[36] Yin X., Dong L., Zheng H., Liu X., Wu H., Yang Y., Ma W., Zhang L., Yin W., Xiao L. (2016). Impact of humidity on quartz-enhanced photoacoustic spectroscopy based CO detection using a near-IR telecommunication diode laser. Sensors.

[37] Wold S., Sjostrom M., Eriksson L. (2001). PLS-regression: A basic tool of chemometrics. Chemom. Intell. Lab. Syst..

[38] Menduni G., Zifarelli A., Sampaolo A., Patimisco P., Giglio M., Amoroso N., Wu H., Dong L., Bellotti R., Spagnolo V. (2022). High-concentration methane and ethane QEPAS detection employing partial least squares regression to filter out energy relaxation dependence on gas matrix composition. Photoacoustics.

[39] Loh A., Wolff M. (2020). Multivariate Analysis of Photoacoustic Spectra for the Detection of Short-Chained Hydrocarbon Isotopologues. Molecules.

[40] Zifarelli A., Patimisco P., Sampaolo A., Giglio M., Menduni G., Elefante A., Vittorio V., Tittel K. F., Spagnolo V. Partial least squares regression as novel tool for gas mixtures analysis in quartz-enhanced photoacoustic spectroscopy. Proceedings of the SPIE, Quantum Sensing and Nano Electronics and Photonics XVII.

[41] Pedregosa F., Varoquaux G., Gramfort A., Michel V., Thirion B., Grisel O., Blondel M., Prettenhofer P., Weiss R., Dubourg V. (2011). Scikit-learn: Machine Learning in Python. JMLR.

[42] Umemura N., Matsuda D., Mizuno T., Kato K. (2014). Sellmeier and thermo-optic dispersion formulas for the extraordinary ray of 5 mol.% MgO-doped congruent LiNbO_3_ in the visible, infrared, and terahertz regions. Appl. Opt..

[43] Schilt S., Thévenaz L., Niklès M., Emmenegger L., Hüglin C. (2004). Ammonia monitoring at trace level using photoacoustic spectroscopy in industrial and environmental applications. Spectrochim. Acta Part A Mol. Biomol. Spectrosc..

